# Morphometric study of Yemeni (*Apis mellifera jemenitica*) and Carniolan (*A*. *m*. *carnica*) honeybee workers in Saudi Arabia

**DOI:** 10.1371/journal.pone.0247262

**Published:** 2021-02-19

**Authors:** Saad N. AL-Kahtani, El-Kazafy A. Taha

**Affiliations:** 1 Arid Land Agriculture Department, College of Agricultural Sciences & Foods, King Faisal University, Alhassa, Saudi Arabia; 2 Economic Entomology Department, Faculty of Agriculture, Kafrelsheikh University, Kafrelsheikh, Egypt; Harran University, TURKEY

## Abstract

The Yemeni honeybee (*Apis mellifera jemenitica* Ruttner) is the native race in Saudi Arabia. The Carniolan honeybee (*A*. *m*. *carnica* Pollmann) and its hybrid with the Egyptian honeybee (*A*. *m*. *lamarkii* Cockerell) have been imported and frequently reared in Saudi Arabia. Temperature often exceed 40 °C during the summer season in most regions of Saudi Arabia. Honeybees decrease foraging activity in this period during mid-day, which affect colony productivity. The Yemeni bee race appears well adapted to these unique climatic conditions. We compared body weight and morphometric parameters of both subspecies’ worker bees reared at the apiary of Training and Research Station, King Faisal University, Al-Ahsa oasis of eastern Saudi Arabia. Measurements of Yemeni bee were smaller than Carniolan bee for body weight, head structures, including antenna, flagellum, and proboscis length, thorax appendages, including femur length, tibia length and width, metatarsus length and width of the right hind leg, and length and width of the right forewing and hind wing, abdominal characteristics, including the length of the 3^rd^ and 4^th^ abdominal tergites and sternites, and length and width of the 1^st^ and 4^th^ wax mirrors. It could be concluded that with the exception of the number of hamuli, worker Yemeni bee body size and morphometric parameters related to the colony productivity were smaller than Carniolan bees under environmental conditions of the study region.

## Introduction

Honeybee (*Apis mellifera* L.) is widespread in Africa, Europe and Western Asia [1], and comprises of 26 subspecies according to morphometric analyses [1–3]. The Yemeni honeybee (*A*. *m*. *jemenitica* Ruttner) is native to Saudi Arabia [2, 4–6]. It occurs naturally in Asia and Africa, including in Sudan and Yemen [7], Chad [8], Oman [9], Saudi Arabia and Somalia [1], Cameroon [10], Ethiopia [11, 12] and Mali [13]. Another subspecies, *A*. *m*. *carnica* Pollmann, has been imported and widely kept in Saudi Arabia [14–20]. However, Yemeni race has been found in areas of highest seasonal temperature and the zone of lowest and most irregular rainfall where other honeybee subspecies are unable to persist [6, 12].

In the semi-arid conditions that cover most parts of Saudi Arabia, temperatures during the summer season often exceed 40 °C, and during this period honeybees not only decrease foraging activity, but also spend a part of time in thermoregulation. Consequently, the productivity of the colony is affected [15]. Overall, Saudi Arabian race of Yemeni bee appears well adapted to unique climatic conditions in Saudi Arabia. For example, their performance in foraging activity, worker and drone brood rearing, storing pollen and colony population size during the hot summer was significantly higher than those of other subspecies [6].

Body characteristics of worker honeybee [12, 21–29] and their comb cell size [30, 31] vary among subspecies. Factors that affect worker body characteristics include pollen and nectar resources [32], content of dietary protein [33, 34], rearing season [35–37], geographical location [38, 39] and level of infestation by the Varroa mite, *Varroa destructor* [37]. In addition, the environmental factors have been shown to affect worker honeybee morphometric characteristics [38, 40, 41].

Body weight is positively correlated with most body characteristics of *A*. *florea* F. [38] and honeybee colony productivity is correlated with body size of worker bee [42, 43]. Body characteristics are used as an indicator of honeybee colony productivity, because workers with larger legs and wings gather higher amounts of nectar and pollen than smaller workers [44]. In particular, honey production is correlated with wing characteristics [43–46], corbicular area [43, 47] and leg characteristics [44, 48].

A comparison of the activity and productivity of local (Yemeni race) and imported bees (Carniolan and Carniolan hybrid) under environmental conditions of Al-Ahsa oasis and Al-Baha region (eastern and southwestern of Saudi Arabia, respectively) have been done. The Yemeni bee colonies showed superiority in stored pollen, worker and drone sealed brood area, colony population size and survival rate [5, 6]. Under the environmental conditions of the Al-Ahsa oasis and the highlands of the Al-Baha region, Yemeni bee colonies produced less honey than the Carniolan and Carniolan hybrid bee colonies [5, 6], while under the lowlands of the Al-Baha region, Yemeni bee colonies produced more honey than the Carniolan hybrid [5].

Here, we compared body weight and morphometric characteristics of Yemeni (native race) and Carniolan (imported race) bee subspecies to find the morphometrical differences between two subspecies under environmental conditions of Al-Ahsa, Saudi Arabia.

## Materials and methods

We measured morphometric parameters of worker bees reared at the apiary of the Training and Research Station, King Faisal University, Al-Ahsa, Saudi Arabia (25°25′46″N, 49°37′19″E; 121 m above sea level) during 2016 alfalfa (*Medicago sativa* L.) flowering season. We obtained ten colonies of Yemeni bee from Gazan (southwest of Saudi Arabia) and ten colonies of Carniolan bee from the Institute for Bee Research, Hohen Neuendorf, Germany during 2015. The colonies were headed by newly mated sister queens and maintained in 8-frame Langstroth hives. We replaced five frames with wax foundation to obtain workers raised on new comb and exclude the effects of comb age.

Nineteen days after queens laid eggs in the comb, worker-sealed brood was inserted into a wooden comb cage (45.25 × 25.35 × 9.45 cm), which was covered with metal gauze, and placed in an incubator set at 32 °C and 60% relative humidity until workers emerged [38]. The fresh body weight (mg) of 50 chilled, newly emerged workers (within 6 h of emergence) was determined using an electrical balance [37] and workers were preserved in 70% ethanol prior to dissection for morphometric analysis [24].

Body appendages were removed, placed on glass slides and measured (mm) under a LABOMED stereomicroscope (Labo America, Inc. USA) equipped with a micrometer lens. From the head, we measured lengths of antenna, flagellum and proboscis. For thorax, we measured femur length, tibia length and width, metatarsus length and width of the right hind leg, length and width of the right forewing and hind wing. We counted the number of hamuli on the right hind wing. For the abdomen, we measured length of the 3^rd^ and 4^th^ abdominal tergites and sternites, and length and width of the 1^st^ and 4^th^ wax mirrors. Measurements were recorded according to Ruttner [2]. We determined the correlations between the body weight and morphometric characteristics to find the relationships between these parameters.

We analyzed subspecies differences in morphometric parameters at *P* = 0.01 using one-way analysis of variance (ANOVA), which indicated significant differences between subspecies. The normality in dataset was tested by the Shapiro-Wilk normality test, which indicated data were normally distributed. Therefore, the analysis was performed on the original data. The ANOVA was used to assess differences between the investigated subspecies, and correlations between characteristics were determined using the PROC GLM function in SAS version 9.1 [49].

## Results

We found that 20 of the 21 examined morphometric parameters significantly (*P* < 0.001) differed between the tested subspecies (Table 1). Compared with the workers of Yemeni subspecies, Carniolan subspecies exhibited significantly (*P* < 0.001) higher values for body weight, total antenna length, flagellum length, proboscis length, length and width of the right forewing and hind wing, femur length, tibia length and width, metatarsus length and width, length of the 3^rd^ and 4^th^ abdominal tergites and sternites, and the length and width of the 1^st^ and 4^th^ wax mirrors. The number of hamuli did not differ between two subspecies.

**Table 1:** Analysis of variance of body weight and morphometric parameters of *Apis mellifera carnica* and *A*. *m*. *jemenitica* workers.

Variable	SS	MS	F value	P value
Body weight	2831.70	2831.70	10453.6	< 0.0001
Proboscis length	3.97	3.97	1110.55	< 0.0001
Flagellum length	1.50	1.50	1018.98	< 0.0001
Total length of antenna	1.65	1.65	1863.90	< 0.0001
Forewing length	6.84	6.84	34626.4	< 0.0001
Forewing width	5.65	5.65	8604.37	< 0.0001
Hindwing length	3.76	3.76	5287.70	< 0.0001
Hindwing width	1.40	1.40	6231.79	< 0.0001
Number of hamuli	0.12	0.12	0.22	> 0.6437
Femur length	0.77	0.77	847.22	< 0.0001
Tibia length	1.39	1.39	15163.9	< 0.0001
Tibia width	0.04	0.04	61.72	< 0.0001
Metatarsus length	1.14	1.14	15049.1	< 0.0001
Metatarsus width	0.06	0.06	1245.86	< 0.0001
3^rd^ tergite length	0.44	0.44	805.40	< 0.0001
3^rd^ sternite length	0.43	0.43	4707.90	< 0.0001
4^th^ tergite length	0.59	0.59	1933.63	< 0.0001
4^th^ sternite length	0.41	0.41	1232.28	< 0.0001
1^st^ Wax mirror length	0.21	0.21	2319.98	< 0.0001
1^st^ Wax mirror width	0.73	0.73	3264.36	< 0.0001
4^th^ Wax mirror length	0.22	0.22	4286.78	< 0.0001
4^th^ Wax mirror width	0.23	0.23	3319.29	< 0.0001

SS = sum of squares, MS = mean squares.

Data presented in Table 3 showed that with the exception of number of hamuli, and width of metatarsus, body weight was positively correlated with morphometric parameters (*r* = 0.68–0.99, *P* < 0.022–0.0001), and proboscis length was correlated with antenna length, forewing length and width, hind wing length and width, tibia and metatarsus lengths, and length and width of 3^rd^ and 4^th^ tergites and sternites, and 1^st^ and 4^th^ wax mirrors (*r* = 0.73–0.99, *P* < 0.01–0.0001). We found that lengths of the 3^rd^ sternite (*r* = 0.92–0.99, *P* < 0.002–0.0001), tibia (*r* = 0.85–0.99, *P* < 0.002–0.0001), and 1^st^ wax mirror (*r* = 0.84–0.99, *P* < 0.002–0.0001) were correlated with morphometrics other than number of hamuli, and width of tibia and metatarsus.

## Discussion

We found that mean body weight of Yemeni bee workers was less than Carniolan bee workers, and these differences were associated with variations in morphometric parameters. Our results confirm the findings of an earlier study that showed Yemeni bee worker had the smallest body size [2]. Intraspecific variation in body size may reflect quality of the environment [50] and worker body weight is altered by the availability of nectar and pollen resources [32, 45, 51], season [35] and colony size [52]. In this study, all colonies were placed in a single location, so it is unlikely that levels of nutrition differed. Moreover, colonies were similar in size; thus, we suggest effects of location or colony size were common to both subspecies.

Among the various structures located on the head, antennae are essential in foraging for nectar and pollen, due to the sensory organs on the flagellum. Honey production is positively correlated with proboscis length, which itself is positively correlated with number of brood [44], and it is known that Carniolan bee colonies produces greater amounts of honey than Yemeni bee colonies [6]. Among the head parameters measured in this study, we found that lengths of proboscis, antenna, and flagellum were shorter in Yemeni bee workers (Table 2), confirming the findings of Alqarni et al. [53] Yemeni bee worker proboscis length in this study was similar to those recorded elsewhere in Saudi Arabia, but shorter than those recorded in Chad, Oman, Somalia, Sudan, and Yemen [54]. These subspecies differences in proboscis, antenna, and flagellum length were correlated with body size, where body weight was positively correlated with proboscis and antenna length (Table 3), supporting findings by Al-Kahtani and Taha [38] for *A*. *florea*.

**Table 2:** Body weights (mg) and morphometric characteristics (mm) of *Apis mellifera carnica* and *A*. *m*. *jemenitica* workers.

Character	Subspecies		Significance
	Carniolan	Yemeni	
Body weight	112.00 ± 6.12	87.65 ± 5.33	**
Proboscis length	6.20 ± 0.56	5.28 ± 0.53	**
Flagellum length	3.14 ± 0.04	2.58 ± 0.05	**
Total length of antenna	4.23 ± 0.06	3.64 ± 0.04	**
Forewing length	9.14 ± 0.12	7.94 ± 0.10	**
Forewing width	3.53 ± 0.02	2.44 ± 0.03	**
Hindwing length	6.74 ± 0.02	5.85 ± 0.01	**
Hindwing width	2.21 ± 0.01	1.67 ± 0.01	**
Number of hamuli	23.10 ± 3.14	23.60 ± 3.06	_NS_
Femur length	2.84 ± 0.03	2.44 ± 0.01	**
Tibia length	3.09 ± 0.02	2.55 ± 0.03	**
Tibia width	1.22 ± 0.01	1.12 ± 0.01	**
Metatarsus length	2.52 ± 0.02	2.03 ± 0.01	**
Metatarsus width	1.21 ± 0.01	1.10 ± 0.01	**
3^rd^ tergite length	2.33 ± 0.01	2.03 ± 0.01	**
3^rd^ sternite length	2.22 ± 0.01	1.92 ± 0.01	**
4^th^ tergite length	2.30 ± 0.01	1.95 ± 0.01	**
4^th^ sternite length	2.22 ± 0.02	1.93 ± 0.01	*
1^st^ Wax mirror length	1.58 ± 0.01	1.37 ± 0.01	*
1^st^ Wax mirror width	2.51 ± 0.01	2.12 ± 0.01	**
4^th^ Wax mirror length	1.46 ± 0.01	1.24 ± 0.01	**
4^th^ Wax mirror width	1.42 ± 0.01	1.20 ± 0.01	**

Values are the mean ± S.D.

**P<0.01 between subspecies,

*P<0.05 between subspecies,

^NS^ P>0.05 between subspecies.

**Table 3:** Pearson correlation coefficients for body weight and morphometric characteristics of *Apis mellifera carnica* and *A*. *m*. *jemeniticsa* workers.

**Characteristics**	**Body weight**	**Proboscis length**	**Flagellum length**	**Antenna length**	**Forewing length**	**Forewing width**	**Hindwing length**	**Hindwing width**	**No. hamuli**	**Femur length**	**Tibia length**	**Tibia width**	**Meta-tarsus length**	**3**^**rd**^ **tergite length**	**3**^**rd**^ **sternite length**	**4**^**th**^ **tergite length**	**4**^**th**^ **sternite length**	**1**^**st**^ **Wax mirror length**	**1**^**st**^ **Wax mirror width**	**4**^**th**^ **Wax mirror length**
**Body weight**																				
**Proboscis length**	0.83**																			
**Flagellum length**	**0.35**	**0.33**																		
**Antenna length**	0.50**	0.40*	0.77**																	
**Fore-wing length**	0.77**	0.69**	**0.31**	0.47*																
**Fore-wing width**	0.74**	0.60**	0.46*	0.64**	0.82**															
**Hind-wing length**	0.60**	**0.35**	**0.34**	0.46*	0.63**	0.67**														
**Hind-wing width**	**0.33**	0.44*	**0.18**	**0.21**	**0.28**	**0.33**	**0.36**													
**No. hamuli**	**0.35**	**0.35**	0.45*	0.50**	**0.36**	**0.38**	0.40*	**0.25**												
**Femur length**	0.50**	**0.35**	**0.33**	0.45*	**0.20**	**0.35**	**0.39**	**0.18**	**0.10**											
**Tibia length**	0.71**	0.42*	0.40*	**0.30**	0.49*	**0.29**	0.58**	0.43*	**0.31**	0.44*										
**Tibia width**	0.62**	**0.35**	0.40*	**0.38**	0.46*	0.63**	0.46*	**0.36**	**0.32**	0.68**	0.75**									
**Metatarsus length**	0.50**	0.53*	0.48*	0.40*	**0.36**	0.68**	0.63**	**0.27**	**0.28**	0.60**	0.50**	**0.36**								
**3**^**rd**^ **tergite length**	**0.38**	**0.22**	0.46*	0.47*	**0.26**	**0.34**	**0.30**	**0.12**	0.40*	0.52**	**0.30**	0.60**	0.40*							
**3**^**rd**^ **sternite length**	0.73**	0.40*	0.53**	0.63**	0.50**	0.75**	0.58**	**0.10**	0.43*	0.57**	**0.33**	0.60**	0.59**	0.68**						
**4**^**th**^ **tergite length**	**0.34**	**0.25**	0.58**	**0.30**	**0.25**	**0.33**	**0.18**	**0.12**	0.41*	**0.10**	**0.31**	0.41*	0.43*	0.50**	0.63**					
**4**^**th**^ **sternite length**	**0.32**	**0.35**	**0.33**	**0.34**	**0.237**	0.42*	0.46*	**0.31**	0.43*	0.40*	0.53**	0.62**	0.62**	**0.38**	0.40*	**0.19**				
**1**^**st**^ **Wax mirror length**	0.63**	0.45*	**0.20**	**0.25**	0.52**	0.65**	0.53**	**0.37**	0.40*	0.41*	0.62**	**0.35**	**0.33**	**0.20**	**0.20**	**0.12**	0.63**			
**1**^**st**^ **Wax mirror width**	0.60**	0.61**	**0.31**	0.45*	0.45*	0.63**	0.52**	0.40*	**0.26**	0.47*	0.69**	0.51**	0.47*	**0.32**	0.42*	**0.16**	**0.37**	0.50**		
**4**^**th**^ **Wax mirror length**	0.61**	0.42*	**0.16**	**0.25**	0.65**	0.66**	0.58**	**0.33**	0.42*	**0.36**	0.65**	**0.33**	**0.35**	**0.19**	**0.21**	**0.10**	0.60**	0.63**	**0.38**	
**4**^**th**^ **Wax mirror width**	0.58**	0.41*	**0.19**	**0.21**	0.58**	0.61**	0.55**	**0.33**	0.44*	**0.37**	0.60**	**0.32**	0.40*	**0.15**	**0.22**	**0.05**	0.59**	0.65**	**0.35**	0.45*

*Correlation is significant at the 0.05 level (2-tailed).

**Correlation is significant at the 0.01 level (2-tailed).

We found that subspecies differed for forewing and hindwing length and width, supporting a study that found honeybee wing size varied with subspecies [55], which can be attributed to differences in body size (Table 2). Body weight was positively correlated with forewing length and width, and hind wing length and width (Table 2), and this has been also reported for *A*. *florea* [38]. Compared with smaller winged bees, bees with larger wings have greater flight power and gather greater amounts of nectar and pollen, resulting in greater potential to rear more brood, and strengthen colony size. Indeed, Mostajeran et al. [44] found positive correlations between brood area and colony size with forewing length and width and hind wing length. A high rate of brood rearing resulted in a larger population size, colony growth, a high rate of colony survival, resulting in increased colony productivity [5, 6, 14].

Measurements of leg parameters were smaller in Yemeni bee workers (Table 2), and body weight was positively correlated with femur, tibia and metatarsus length (Table 3), similar to findings obtained for *A*. *florea* [38]. Bees with longer legs may gather higher amounts of pollen, resulting in increases in brood and colony size and colony productivity. It has been shown that honey production is correlated with wing and leg characteristics [44, 46] and corbicular area [43, 47]. These results explain the greater honey production of Carniolan bee colonies than Yemeni bee colonies [6]. In the meantime, Yemeni bee race gather greater amounts of pollen, rear more brood and increase colony size compared to imported bees [5, 6].

There were differences in the lengths of 3^rd^ and 4^th^ tergites and sternites, and 1^st^ and 4^th^ wax mirrors between the two subspecies (Table 2). These differences were correlated with worker body size. For example, body weight was correlated with length of the 3^rd^ and 4^th^ tergites, 3^rd^ and 4^th^ sternites, and 1^st^ and 4^th^ wax mirrors, and width of the 1^st^ and 4^th^ wax mirrors (Table 3), supporting similar observations for *A*. *flora* [38]. We noted that length of the 3^rd^ sternite in Yemeni bee workers was shorter than Yemeni bee workers from west and north-eastern Africa [13] and Ethiopia [12]. The lengths of the 3^rd^ and 4^th^ tergites, and 3^rd^ and 4^th^ sternites refer to the length and width of the abdomen and the size of the honey stomach, which affect the amount of gathering of nectar and honey yield [32, 56]. The dimensions of wax mirrors have been reported as an important factor affect building of the combs [57].

## Conclusions

With the exception of the number of hamuli, data from this study showed that body size and morphometric parameters related to the colony productivity of worker Yemeni bee (*A*. *m*. *jemenitica*) were smaller than worker Carniolan bees (*A*. *m*. *carnica*) in Saudi Arabia. Body weight can be used as an indicator of body size and morphometric parameters related to colony productivity.

## References

[1] Miguel I, Baylac M, Iriondo M, Manzano C, Garnery L, Estonba A. Both geometric morphometric and microsatellite data consistently support the differentiation of the *Apis mellifera* M evolutionary branch. Apidologie. 2011;42: 150–161.

[2] Ruttner F. Biogeography and taxonomy of honeybees. Berlin, Heidelberg, New York: Springer-Verlag; 1988.

[3] Engel M. The taxonomy of recent and fossil honeybees. J Hymenopt Res. 1999;8: 165–196.

[4] Taha EKA, Alqarni AS. Morphometric and reproductive organs characters of *Apis mellifera jemenitica* drones in comparison to *Apis mellifera carnica*. Int J Sci Eng Res. 2013;4: 411–415.

[5] Al-Ghamdi AA, Adgaba N, Tadesse Y, Getachew A, Al-Maktary AA. Comparative study on the dynamics and performances of *Apis mellifera jemenitica* and imported hybrid honeybee colonies in southwestern Saudi Arabia. Saudi J Biol Sci. 2017;24: 1086–1093. 10.1016/j.sjbs.2017.01.008 28663709 PMC5478372

[6] Taha EKA, Al-Kahtani SN. Comparison of the activity and productivity of Carniolan (*Apis mellifera carnica* Pollmann) and Yemeni (*Apis mellifera jemenitica* Ruttner) subspecies under environmental conditions of the Al-Ahsa oasis of eastern Saudi Arabia. Saudi J Biol Sci. 2019;26: 681–687. 10.1016/j.sjbs.2017.10.009 31048992 PMC6486514

[7] Ruttner F. African races of Honeybees. In: Proceedings, 25th international beekeeping congress. Grenoble, France; 1976. pp. 325–344.

[8] Gadbin C, Cornuet JM, Fresnaye J. Approche biométrique de la variété locale d’ *Apis mellifica* L. Dans le sud tchadien. Apidologie. 1979;10: 137–148.

[9] Dutton RW, Ruttner F, Berkeley A, Manley MJD. Observations on the morphology, relationships and ecology of *Apis mellifera* of Oman. J Apic Res. 1981;20: 201–214.

[10] Meixner M, Ruttner F, Koeniger N, Koeniger G. The mountain bees of the Kilimanjaro region and their relation to neighbouring bee populations. Apidologie. 1989;20: 165–174.

[11] Radloff SE, Hepburn HR. Multivariate analysis of honeybees, *Apis mellifera* Linnaeus (Hymenoptera: Apidae), of the Horn of Africa. Afr Entomol. 1997;5: 57–64.

[12] Amssalu B, Nuru A, Radloff SE, Randall Hepburn H. Multivariate morphometric analysis of honeybees (*Apis mellifera*) in the Ethiopian region. Apidologie. 2004;35: 71–81.

[13] Hepburn HR, Radloff SE. Honeybees of Africa. Berlin, Germany,: Springer Berlin Heidelberg; 1998.

[14] Taha EKA, Al-Kahtani SN. Relationship between population size and productivity of honeybee colonies. J Entomol. 2013;10: 163–169.

[15] Taha EKA. Seasonal variation of foraging activity, pollen collection and growth of honeybee colonies in Al-Ahsa, Saudi Arabia. Bull Entomol Soc Egypt. 2014;91: 163–175.

[16] Al-Kahtani SN, Taha EKA, Al-Abdulsalam M. Alfalfa (*Medicago sativa* L.) seed yield in relation to phosphorus fertilization and honeybee pollination. Saudi J Biol Sci. 2017;24: 1051–1055. 10.1016/j.sjbs.2016.12.009 28663703 PMC5478283

[17] Taha EKA, Al-Abdulsalam M, Al-Kahtani S. Insect pollinators and foraging behavior of honeybees on alfalfa (*Medicago sativa* L.) in Saudi Arabia. J Kans Entomol Soc. 2016;89: 92–99.

[18] Taha EKA, Al-Kahtani S, Taha R. Protein content and amino acids composition of bee-pollens from major floral sources in Al-Ahsa, eastern Saudi Arabia. Saudi J Biol Sci. 2019;26: 232–237. 10.1016/j.sjbs.2017.06.003 31485159 PMC6717098

[19] Al-Kahtani SN, Taha EKA. Post grafting time significantly influences royal jelly yield and content of macro and trace elements. Plos One 2020;15: e0238751 10.1371/journal.pone.0238751 32898187 PMC7478805

[20] Al-Kahtani SN, Taha EKA, Khan KhA, Ansari MJ, Farag SA, Shawer DMB, et al Effect of harvest season on the nutritional value of bee-pollen protein. Plos One 2020;15: e0241393 10.1371/journal.pone.0241393 33370277 PMC7769433

[21] Milne CP, Hellmich RL, Pries KJ. Corbicular size in workers from honeybee lines selected for high or low pollen hoarding. J Apic Res. 1986;25: 50–52.

[22] Atallah MA, Aly FK, Eshab MH. Comparative morphometrical investigations of the Egyptian, Carniolan and Italian honeybee races in Minia region. J Agric Res. 1987;9: 1307–1319.

[23] Oldroyd B, Rinderer T, Buco S. Heritability of morphological characters used to distinguish European and Africanized honeybees. Theor Appl Genet. 1991;82: 499–504. 10.1007/BF00588605 24213268

[24] Adl MB, Gencer HV, Firati C, Bahreini R. Morphometric characterization of Iranian (*Apis mellifera meda*), central Anatolian (*Apis mellifera anatoliaca*) and Caucasian (*Apis mellifera caucasica*) honeybee populations. J Apic Res. 2007;46: 225–231.

[25] Alburaki M, Aalburaki A. Morphometrical study on Syrian honeybee (*Apis mellifera syriaca*). Emir J Food Agric. 2008;20: 89–93.

[26] Ozbakir GO, Firatli C. Morphometric classification of honeybee populations (*Apis mellifera* L.) along the southeast border of Turkey. Bulg J Agric Sci. 2013;19: 1396–1400.

[27] Alattal Y, Al Ghamdi A, Al Sharhi M, Fuchs S. Morphometric characterisation of the native Honeybee, *Apis mellifera* Linnaeus, 1758, of Saudi Arabia. Zool Middle East. 2014;60: 226–235.

[28] Oleksa A, Tofilski A. Wing geometric morphometrics and microsatellite analysis provide similar discrimination of honeybee subspecies. Apidologie. 2015;46: 49–60.

[29] Amakpe F, De Smet L, Brunain M, Jacobs FJ, Sinsin B, de Graaf DC. Characterization of native honeybee subspecies in republic of benin using morphometric and genetic tools. J Apic Sci. 2018;62: 47–60.

[30] Abdellatif MA. Comb cell size and its effect on the body weight of the worker bee, *Apis mellifera* L. Am Bee J. 1965;105: 86–87.

[31] Al-Kahtani SN. Morphometrical characteristics of Carniolan honeybee workers in relation to age of comb. Sci J King Faisal Uni. 2018;19: 47–54.

[32] Helal RM, El-Dakhakhni TN, Shawer MB, Taha EKA. Effect of moving the apiaries on activity of honeybee colonies. 2- flight activity, gathering of nectar and sugar concentration contents and honey. J Agric Res Tanta Univ. 2003;29: 268–282.

[33] Roulston TH, Cane JH. Pollen nutritional content and digestibility for animals. Plant Syst Evol. 2000;222: 187–209.

[34] Zheng B, Wu Z, Xu B. The effects of dietary protein levels on the population growth, performance, and physiology of honeybee workers during early spring. J Insect Sci. 2014;14: 1–17. 25368092 10.1093/jisesa/ieu053 PMC5443605

[35] Kunert K, Crailsheim K. Seasonal changes in carbohydrate, lipid and protein content in emerging worker honeybees and their mortality. J Apic Res. 1988;27: 13–21.

[36] Ivanov T, Spasov K. Study of some physiological parameters characterizing productivity and winter resistance of honeybee. Zhivotn Nauki. 1990;26: 67–73.

[37] Kovac H. Dry weight of newly emerged worker honeybees (*Apis mellifera carnica* Pollm.) in dependence on infestation with the Varroa mites (*Varroa jacobsoni* Oud.) and on season. Mitt Dtsch Ges fuer Allg Angew Entomol. 1993;8: 749–753.

[38] Al-Kahtani SN, Taha EKA. Morphometric studies on dwarf honeybee *Apis florea* F. workers in Saudi Arabia. J Apic Sci. 2014;58: 127–134.

[39] Kuliçi M, Kume K. Study on morphometric traits of the Albanian bees. Albanian J Agric Sci. 2014;(Special edition): 419–425.

[40] Ajao AM, Oladimeji YU, Idowu AB, Babatunde SK, Obembe A. Morphological characteristics of *Apis mellifera* L. (Hymenoptera: Apidae) in Kwara State, Nigeria. Int J Agric Sci. 2014;4: 171–175.

[41] Charistos L, Hatjina F, Bouga M, Mladenovic M, Maistros AD. Morphological discrimination of greek honeybee populations based on geometric morphometrics analysis of wing shape. J Apic Sci. 2014;58: 75–84.

[42] Waddington KD. Implications of variation in worker body size for the honeybee recruitment system. J Insect Behav. 1989;2: 91–103.

[43] Kolmes SA, Sam Y. Relationships between sizes of morphological features in worker honeybees (*Apis mellifera*). J N Y Entomol Soc. 1989;99: 684–690.

[44] Mostajeran M, Edriss MA, Basiri MR. Analysis of colony and morphological characters in honeybees (*Apis mellifera meda*). Pak J Biol Sci. 2006;9: 2685–2688.

[45] Szabo TI, Mueller AE. Factors affecting the weight changes of honeybee colonies. Am Bee J. 1996;136: 417–419.

[46] Edriss MA, Mostajeran M, Ebadi R. Correlation between honey yield and morphological traits of honeybee in Isfahan. J Sci Technol Agric Nat Resour. 2002;6: 91–103.

[47] Milne CP, Pries KJ. Honeybee corbicular size and honey production. J Apic Res. 1984;23: 11–14.

[48] Szabo TI, Lefkovitch LP. Fourth generation of closed population honeybee breeding. 2. Relationship between morphological and colony traits. Apidologie. 1988;19: 259–274.

[49] Sas S, Guide SUs. Version 9.1. SAS Institute Inc, Cary, NC. 2003.

[50] Banaszak-Cibicka W, Fliszkiewicz M, Langowska A, Żmihorski M. Body size and wing asymmetry in bees along an urbanization gradient. Apidologie. 2018;49: 297–306.

[51] Szentgyörgyi H, Czekońska K, Tofilski A. Influence of pollen deprivation on the fore wing asymmetry of honeybee workers and drones. Apidologie. 2016;47: 653–662.

[52] Calatayud F, Verdu JM. Evolución anual de parámetros poblacionales de colonias de *Apis mellifera* L. (Hymenoptera: Apidae) parasitadas por *Varroa jacobsoni* Oud. (Mesostigmata: Varroiadae). Bol San Veg Plagas. 1992;18: 777–788.

[53] Alqarni AS, Hannan MA, Owayss AA, Engel MS. The indigenous honeybees of Saudi Arabia (Hymenoptera, Apidae, *Apis mellifera jemenitica* Ruttner): their natural history and role in beekeeping. ZooKeys. 2011;134: 83–98. 10.3897/zookeys.134.1677 PMC3229212 22140343

[54] Al-Ghamdi AA, Nuru A, Khanbash MS, Smith DR. Geographical distribution and population variation of *Apis mellifera jemenitica* Ruttner. J Apic Res. 2013;52: 124–133.

[55] Barour C, Baylac M. Geometric morphometric discrimination of the three African honeybee subspecies *Apis mellifera intermissa*, *A*. *m*. *sahariensis* and *A*. *m*. *capensis* (Hymenoptera, Apidae): Fore wing and hind wing landmark configurations. J Hymenopt Res. 2016;52: 61–70.

[56] Taha EKA. Effect of transferring the apiaries on activity of honeybee colonies. Unpublished M. Sc. Thesis, Tanta University. 2000.

[57] Taha EKA. Studies on honeybee (Apis mellifera L.). Unpublished Ph.D. Thesis, Tanta University. 2005.

