# Expression of Concern: Morphometric study of Yemeni (*Apis mellifera jemenitica*) and Carniolan (*A*. *m*. *carnica*) honeybee workers in Saudi Arabia

**DOI:** 10.1371/journal.pone.0284678

**Published:** 2023-04-19

**Authors:** The PLOS ONE Editors

After this article [1] was published, it was identified as being linked to a series of submissions for which PLOS identified concerns about authorship and potential competing interests. The concerns have implications for the integrity of this article’s peer review and the article’s adherence to PLOS policies, but *PLOS ONE* concluded that the evidence pertaining to this article was inconclusive.

PLOS reassessed the article with input from an Editorial Board member, and noted concerns about this article’s contribution to the field given the study design and other published work that addressed similar research questions (e.g. [2–4]).

The *PLOS ONE* Editors issue this Expression of Concern to notify readers of the above issues.

The corresponding author noted that there are errors in Table 2: the significance markings (column 4) for both the 4^th^ sternite length and 1^st^ Wax mirror length should be **.

The data underlying this article’s results are in S1 File.

## Supporting information

S1 File Study dataset. (XLSX)
